# The Continuity of Living Matter

**Published:** 1898-02

**Authors:** 


### The Continuity of Living Matter.

Prof. Rudolph Virchow, at the twelfth International Medical Congress, lately held at Moscow, reiterated with warmth in an opening address his views of cellular life. "Life," he said, "has no other origin than from life itself. Life is in the cell. He who speaks of serum as a vital force apart from cells is wrong. The grand truth of cellular succession may be assailed in the future, as it has been in the past, but it will never be thrown to earth."

